# Environmental Effects on the Performance of Quantum Dot Luminescent Solar Concentrators

**DOI:** 10.1021/acsphotonics.3c00788

**Published:** 2023-07-28

**Authors:** Meghna Siripurapu, Francesco Meinardi, Sergio Brovelli, Francesco Carulli

**Affiliations:** † Indian Hill School, 6855 Drake Road, Cincinnati, Ohio 45243, United States; ‡ Dipartimento di Scienza dei Materiali, Università degli Studi di Milano-Bicocca, Via Cozzi 55, Milan 20126, Italy; § Glass to Power, via Fortunato Zeni 8, Rovereto I-38068, Trento, Italy

**Keywords:** luminescent solar concentrators, quantum dots, environmental effect, building-integrated photovoltaics, optical waveguides

## Abstract

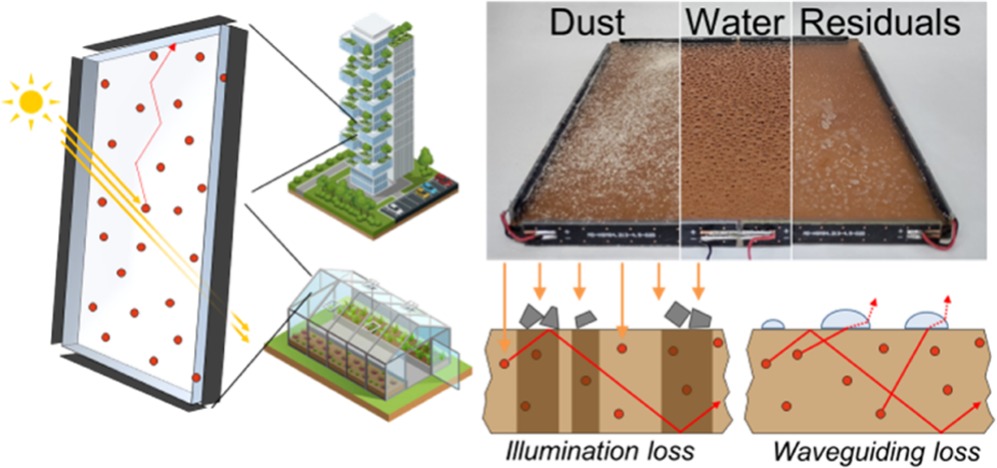

Luminescent solar concentrators (LSCs) are all-photonic, semitransparent solar devices with great potential in the emerging fields of building-integrated photovoltaics and agrivoltaics. Over the past decade, particularly with the advent of quantum dot (QD) LSCs, tremendous progress has been made in terms of photovoltaic efficiency and device size by increasing solar spectral coverage and suppressing reabsorption losses. Despite these advances in LSC design, the effects of environmental conditions such as rain, dust, and dirt deposits, which are ubiquitous in both urban and agricultural environments, on LSC performance have been largely overlooked. Here, we address these issues by systematically investigating the environmental effects on the solar harvesting and waveguiding capability of state-of-the-art QD-LSCs, namely, the presence of airborne pollutants (dust), water droplets, and dried deposits. Our results show that dust is unexpectedly insignificant for the waveguiding of the concentrated luminescence and only reduces the LSC efficiency through a shadowing effect when deposited on the outer surface, while dust accumulation on the inner LSC side increases the output power due to backscattering of transmitted sunlight. Water droplets, on the other hand, do not dim the incident sunlight, but are detrimental to waveguiding by forming an optical interface with the LSC. Finally, dried deposits, which mimic the evaporation residues of heavy rain or humidity, have the worst effect of all, combining shading and waveguide losses. These results are relevant for the design of application-specific surface functionalization/protection strategies real LSC modules.

## Introduction

**Figure 1:**
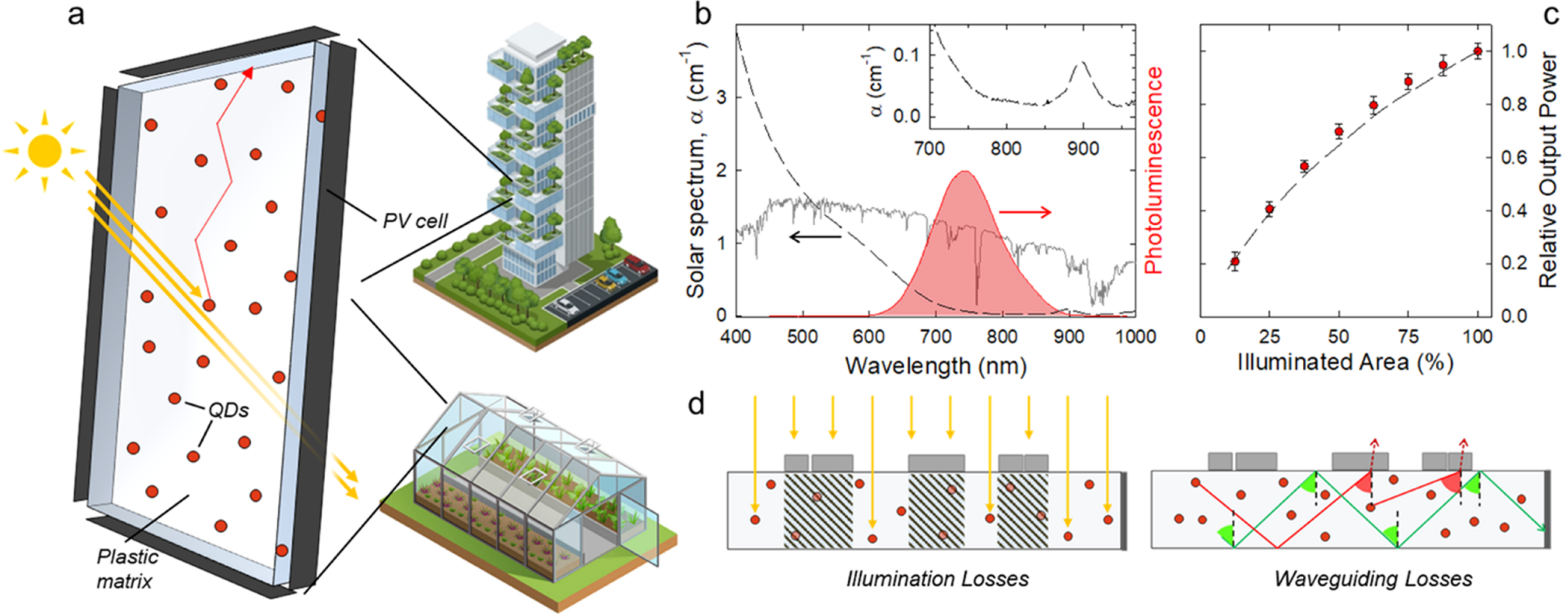
(a) Sketch of a QD-LSC composed of a polymer slab coupled to PV cells and its application in an urban context or in agriculture as energy-producing greenhouse panels. (b) Optical absorption (black dashed line) and PL (red shaded line) spectra of CuInS_2_/ZnS QDs in PMMA LSC. Solar irradiance in AM 1.5 conditions (grey line). (c) Relative optical power output measured from the LSC edge as a function of device area illuminated by a calibrated solar simulator (red circles). The Monte Carlo simulation for an ideal LSC is shown as a dashed curve. (d) Schematic representation of the loss mechanisms studied in this work.

In recent years, awareness of the adverse effects of climate change, coupled with growing concerns about energy supply, has led governments to increasingly promote the transition to renewable energy technologies in a wide range of sectors, from green mobility to sustainable architecture.^1,2^ In the latter area, several countries have already adopted stringent requirements for new buildings to be near-zero energy buildings (NZEBs), requiring both the use of energy-efficient materials and the incorporation of energy-generating technologies into the built environment, which is the core of so-called building-integrated photovoltaics (BIPV).^3−6^ Luminescent solar concentrators (LSCs) are of particular interest for the integration of semitransparent PV devices into the envelope of glass buildings.^7−11^ Specifically, LSCs consist of plastic or glass waveguides containing highly luminescent chromophores that absorb a fraction of the incident solar radiation and emit lower-energy photons that are concentrated by total internal reflection at the waveguide edges, where small PV cells convert them into electrical energy (Figure 1a).^12^ Crucially, unlike other BIPV approaches, the fully photonic operating mechanism of LSCs does not require electrodes to be placed on the device surfaces. As a result, LSCs are arguably the only technology capable of producing semitransparent PV glazing that is both aesthetically pleasing and does not interfere with the view from inside to outside.^7,13−16^ In addition, the light weight and design versatility of LSCs in terms of color and transparency make them particularly promising for applications in so-called agrivoltaics for the realization of self-powered greenhouses with increased mass production through crop-specific spectral tuning of the transmitted sunlight by the LSC cover.^17−21^

After a few decades of apparent waning interest, the advent of colloidal semiconductor quantum dots (QDs) as reabsorption-free NIR LSC emitters nearly a decade ago has revived research in the field,^22−31^ leading to significant advances in power efficiency and device size, both of which are essential for real-world implementation.^26,28,32−34^ Important advances have been made in the design of so-called Stokes-shift-engineered QDs with large spectral separation between their absorption and photoluminescence (PL) spectra and in the development of industrial-scale fabrication protocols for QD-LSC waveguides.^26,28,34−37^ To date, the highest efficiencies have been achieved with I–III–VI_2_ QDs such as CuInS_2_ and related heterostructures (e.g., CuInS_2_/ZnS), which have a natural wide Stokes shift and NIR PL,^26,28,35,38−40^ although important advances have also been demonstrated with binary chalcogenides^41−44^ and metal halides.^45−48^ Despite this progress in the design of LSC waveguides, very little has been done toward a real-world implementation of this technology, which necessarily involves an assessment of the impact of environmental factors on LSC performance,^49−53^ which ultimately determines the relevance and type of encapsulation/protection required for operation in a real-world context. A compelling example of this is the effect of various types of dry or wet deposits on an LSC waveguide, which in addition to reducing the amount of solar light that can reach the waveguide and be converted into useful guided PL—as it commonly occurs for direct charge generation in conventional PV modules^54^—can also affect the transport of light energy to the device edges by disrupting the waveguiding by total internal reflection.

In this work, we aim to contribute to this endeavor by investigating the impact on LSC performance of probably the most common environmental factors that could occur in urban or agricultural contexts, namely, dust accumulation, wetting by water droplets, or the presence of continuous wet layers on the external or internal surfaces of the panels, as well as the influence of dried residues left after evaporation of water, as could occur after rainfall on buildings or irrigation in greenhouses. The quantitative performance evaluation indicated that the accumulation of dust on the LSC outer surface reduces the LSC efficiency by a shadowing effect that lowers the intensity of sunlight entering the panel (similar to what commonly happens to conventional PV modules) but does not detriment the waveguiding behavior, leading to no losses of propagating luminescence. On the other hand, the continuous optical interface between the surface of a polyacrylate LSC and water droplets was found to be very detrimental to light propagation. Finally, the accumulation of dried residues combined the detrimental effects of dust and liquid deposits, causing both shadowing of the LSC and reduced waveguiding. These results provide some clear guidelines for rationalizing the future treatments on LSC surfaced designed to preserve their performances.

## Results and Discussion

Large-area poly(methyl methacrylate) (PMMA) LSC waveguides (20 × 20 cm^2^, thickness = 7.5 mm, 70% transmittance between 400 and 900 nm) containing CuInS_2_/ZnS QDs (0.1 wt %) were fabricated by industrial cell casting of methyl methacrylate monomers using Lauroyl peroxide as thermal radical initiator (500 ppm).^38^ The QDs were prepared according to the synthetic protocol described in the Methods section of Supporting Information, and their size was tuned to provide maximum spectral coverage while matching the transparency window of the LSC, which is determined by the absorption onset of the QDs (short wavelength limit) and the first C–H absorption overtone of the PMMA at ∼915 nm (long wavelength limit). The absorption and PL spectra of the LSC are shown in Figure 1b together with the AM 1.5 solar spectrum; the PL quantum yield of the QDs in the waveguide was Φ_PL_ = 65%.

The suppression of self-absorption by the QDs and the high optical quality of the PMMA waveguide led to an essentially complete absence of reabsorption and scattering losses, as confirmed by the inset of Figure 1b and the relative output power values vs fraction of illuminated device area reported in Figure 1c. In particular, the experimental data, corroborated by Monte Carlo ray-tracing simulations, showed that the fractions of the QD-LSC participated to the total output power almost identically to that expected from an ideal absorption- and scattering-free LSC—where the contributions by illuminated portions at different distance from the perimeter PV cells are due to geometrical factors—in good agreement with previous reports on similar LSCs based on CuInS_2_ QDs^28^ (details of the simulation procedure are reported in the Methods section in the Supporting Information).

**Figure 2:**
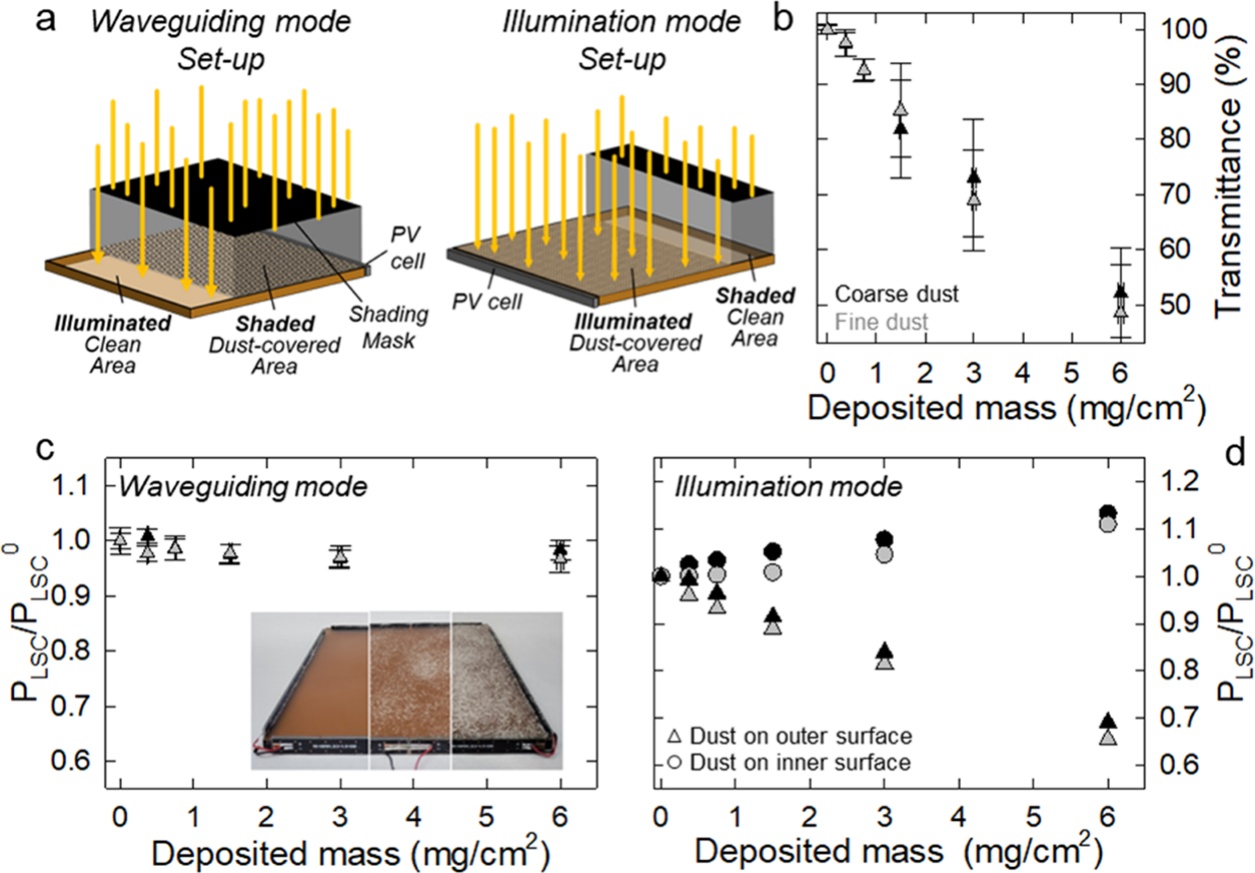
(a) Schemes of the experimental configurations used. (b) Optical transmittance as a function of the amount of coarse (black symbols) and fine (gray symbols) dust grains deposited on the LSC surface. The same color scheme is applied across the figure. (c) Relative power output extracted from one edge of LSC (20 × 20 cm^2^) measured in waveguiding mode as a function of the surface coverage. Inset: photographs of the tested LSC with increasing surface coverage. (d) Relative power output extracted from an LSC as a function of the surface coverage measured in illumination mode with dust on the outer (triangles) or on the inner (circles) LSC face. The error bars are the standard deviation calculated over eight repetitive measurements. In plot (d), the error bars are within the size of the data points.

Having verified the quality of our testbed QD-LSC device, we proceeded to investigate the effect of different types of deposit in order to reproduce real operating conditions. In these experiments, we focused on the two processes that most determine the performance of LSCs, sunlight harvesting, and light propagation. As shown schematically in Figure 1d, the presence of light-absorbing or scattering deposits on the LSC surface could act as a shading agent, partially reducing the illuminated device area, similar to what commonly occurs in conventional PV modules. In addition, if the LSC surface and accumulated deposits (with similar or higher refractive index) form an optical interface, the waveguiding capability of the device could be dramatically degraded by large local photon escape cone losses (Figure 1d, right). To address these effects independently, we designed two different experimental configurations shown in Figure 2a. The first configuration, called *waveguiding mode*, targeted the light-guiding performance of an LSC and consisted of illuminating the terminal part of an LSC, which was kept clean, with a calibrated solar simulator, while the majority of the device surface was covered with increasing amounts of deposits (dust, water, residues) and kept in darkness by a shadow mask.

This allowed us to keep the power absorbed by the LSC constant while monitoring the effect of the deposits on the propagation of the generated PL. In the second configuration, referred to as the *illumination mode* (Figure 2b), the clean part of the same LSC was kept in the dark by moving the shadow mask and the part with the deposits was directly illuminated by the simulated sunlight. This latter experiment provided information on both propagation and illumination effects, which were decoupled by the comparative analysis of the two experimental modes. In both cases, the output power was measured from one LSC edge only, whereas Si PV cells were coupled to all four sides as in real LSC devices. We first reproduced the effect of sand/dust accumulation by progressively covering the outer surface of the LSC waveguide with increasing amounts of dust powder. Surface coverage was quantified by measuring the intensity of the light transmitted through the LSC at five independent points (Figure 2b, from 100 to 50% corresponding to up to 6 mg/cm^2^) and was found to correlate linearly with the mass amount of deposited material, suggesting that in the mass range analyzed and with the covering method adopted, the artificial dust powder created an essentially single layer on progressively larger fractions of the LSC panel. By operating in waveguiding mode with powder samples of two different grain sizes (<100 μm, refer to as fine dust, and >200 μm, coarse dust), we observed that the relative power collected from the LSC edge was nearly independent of the surface coverage (Figure 2c), indicating that the accumulation of dust had essentially no effect on light propagation, probably due to the low optical coupling at the LSC/dust interface, which could not outcouple guided photons. Considering the relatively large amounts of artificial dust used in the experiment (up to 6 mg/cm^2^), this behavior was positive for the LSCs, as it suggested that the device operation tolerated significant levels of dust contamination. On the other hand, the presence of dust was critical for the solar harvesting capability of the LSC (as expected given the decrease of as much as 50% in transmitted light shown in Figure 2b), leading to a progressive decrease in relative power output (up to 35%) with increasing surface coverage (Figure 2d, triangles). Note that the decrease in relative power output was less than the decrease in transmittance (35 vs 50%). This could be explained by two facts: (i) the simulated sunlight incident perpendicularly on the LSC surface was partially scattered by the dust grains, resulting in longer propagation paths inside the LSC panel, artificially increasing its absorbance, and/or (ii) the surface dust acted as a back reflector for the transmitted solar light (note that the LSC had an absorbance of ∼30% between 400 and 900 nm), in both cases artificially increasing the relative power output. The second scenario was confirmed by performing the waveguide mode experiment with the dust deposited on the inner surface of the LSC (on the opposite side of the solar simulator), which showed 12–15% greater relative power output as the surface coverage increased (Figure 2d, circles). In this case, the dust clearly acted as a back scatterer for photons transmitted through the device, artificially increasing the solar irradiance. It is worth noting that such increased relative power output was not due to scattering of transmitted sunlight directly onto the perimeter PV cells, as light generated outside the LSC waveguide (as in the case of light scattered by the dust deposit) would not be guided by total internal reflection and would therefore only propagate 6–7 mm (due to geometrical factors) inside the LSC before exiting the waveguide.

**Figure 3:**
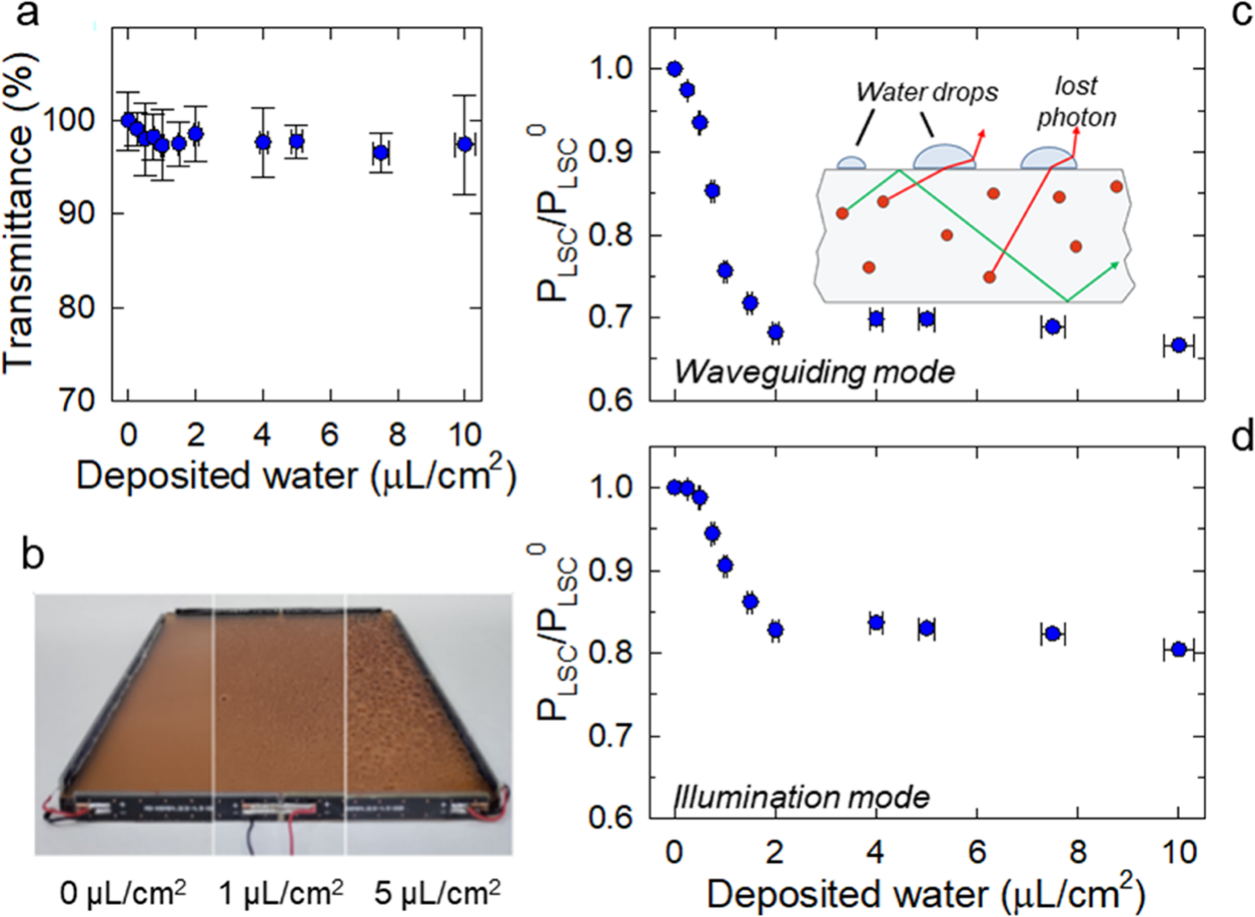
(a) Photographs of an LSC with increasing wetting level. (b) Light transmittance (400–900 nm) as a function of the wetting level. Relative power output extracted from one edge of the QD-LSC (20 × 20 cm^2^) as a function of wetting level measured (c) in waveguiding mode and (d) in illumination mode. The error bars are the standard deviation calculated over eight repetitive measurements.

We then reproduced the effect of raindrops or condensed moisture by spraying the surface of the same LSC with increasing amounts of deionized water (Figure 3a). In this case, the light transmission was unaffected by the deposited water (expressed by the “wetting level” up to 10 μL/cm^2^, Figure 3b), which is consistent with the low absorption of water in the vis–NIR spectral region (Supporting Figure S), and therefore the power output from the LSC was expected to be little affected by shadowing effects. In contrast, the optical interface between the liquid and the PMMA waveguide resulted in severe waveguiding losses, as illustrated in Figure 3c, which shows a drop in relative output power of approximately 35% at a wetting level of 2 μL/cm^2^. Above this level, the output power saturated, probably due to the agglomeration of water droplets into a continuous layer whose dimensions grew by a negligible amount with further water deposition, resulting in an essentially constant average effect. Consistent with the strong perturbation of the waveguiding behavior of the LSC and the negligible effect of the wetting layer on the light transmittance, a very similar trend of relative output power with wetting level was found for the illumination mode experiments (Figure 3d).

**Figure 4:**
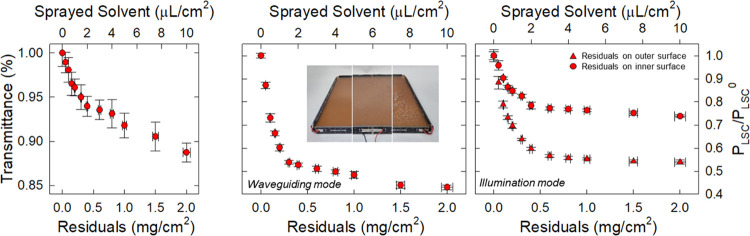
(a) Light transmittance as a function of the surface coverage. Relative power output extracted from one edge of the QD-LSC (20 × 20 cm^2^) as a function of the surface coverage measured (b) in “waveguiding mode” and (c) in “illumination mode”. Inset: photograph of the QD-LSC with increasing surface coverage by dried residues. The error bars are the standard deviation calculated over eight repetitive measurements.

Finally, we tested the effect of dried residues from heavy water evaporation by depositing and evaporating a highly concentrated aqueous solution of NaCl (200 g/L) on the LSC panel. In this case, the overall effect was intermediate between the dust and water situations described above: increasing the amount of dried residue resulted in a gradual decrease in device transmittance, consistent with the presence of light-scattering NaCl domains on the device surface (Figure 4a), similar to that observed with dust deposits. Crucially, and consistent with the effect of water, the relative output power dropped significantly at relatively low surface coverages, consistent with the formation of an optical coupling between the residues and the LSC panel, with an initial strong effect of small droplets on light propagation followed by an average effect of large wetting layers and associated residues. The reduced waveguiding capability in the presence of dried residues (up to 60% of the initial value) also had a dramatic effect on the relative output power measured in illumination mode. It should be emphasized that, as in the case of deionized water, the loss in illumination mode was lower (∼45%) than in waveguiding mode due to the shorter average distance traveled by the photons in the latter configuration. Interestingly, when we performed the illumination mode experiment with dried residues on the inner surface of the LSC, we found significantly lower losses, consistent with a backscattering effect of the transmitted light that partially mitigated the loss due to perturbed waveguiding.

## Conclusions

In conclusion, we have studied the effects of different types of environmental agents, representing real cases in both BIPV and agrivoltaic use of LSCs, on the performance of state-of-the-art QD-LSCs, specifically dust, water, and dried residues, separately considering their effects on solar harvesting and waveguiding behavior. We found that dust deposits have little effect on the waveguiding performance but reduce the power output essentially in proportion to their mass amount (when directly proportional to the consequent surface coverage). Dust deposits on the inner surface, on the other hand, increase the power output by backscattering transmitted light into the LSC. The most limiting factor for light propagation was found to be the presence of wetting layers or dried residues forming optical interfaces with the LSC matrix, the latter causing a combination of the loss effects of dust and moisture. These results provide important insights into the parasitic process affecting the behavior of LSCs and suggest that specific surface functionalization approaches could be implemented to favor the integration of LSCs without bulky encapsulation, such as the use of self-cleaning highly hydrophobic coatings.

## Supporting Information Available

Detailed description of the QDs synthesis and fabrication of LSC; experimental methods; and a detailed description of Monte Carlo ray-tracing simulation (PDF)
