# Supplementary material for: Environmental Effects on the Performance of Quantum Dot Luminescent Solar Concentrators
Source: ACS Photonics. 2023 Jul 28;10(8):2987–93. doi: 10.1021/acsphotonics.3c00788 (PMC10436347; doi:10.1021/acsphotonics.3c00788)
Supplement: Supplementary file 1 — ph3c00788_si_001.pdf [file ph3c00788_si_001.pdf]

Supporting Information

Environmental effects on the performance of Quantum-Dot luminescent solar concentrators

Meghna Siripurapu, Francesco Meinardi, Sergio Brovelli and Francesco Carulli

Corresponding authors:

francesco.carulli@unimib.it
sergio.brovelli@unimib.it

Methods

Chemicals - Copper(I) iodide (CuI, ≥99.5%), indium(III) acetate (In(OAc)3, 99.99%), Zinc Stereate ($Zn(St)_2$ technical grade, 90% as a mixture of C-16 and C-18 isomers) 1-dodecanethiol (DDT, ≥98%), oleic acid (OA, ≥90%), and 1-octadecene (ODE, ≥90%) were purchased from Sigma-Aldrich. Hexane (chromasolv, ≥97%), acetone (puriss. ≥99%) and ethanol (puriss. ≥99%), were purchased from Honeywell Riedel-de-Haën. All the chemicals were used without further purification. Methylmethacrylate (MMA, 99%, Aldrich), purified with basic activated alumina (Sigma-Aldrich), was used as a monomer for the preparation of polymeric nanocomposites. Lauroyl peroxide (98%, Aldrich) and 2,2′-azobis(2-methylpropionitrile) (AIBN, 98%, Aldrich) were used as initiators without purification.

Synthesis of $CuInS_2$/ZnS core/shell QDs - The synthesis of $CuInS_2$ QDs was performed following a heat-up procedure. A mixture of CuI (0.4 mmol), $In(OAc)_3$ (0.4 mmol), and 5 mL DDT was loaded into a 3-necked flask and degassed under vacuum at 130 °C for 1 h. To grow particles of different sizes, the temperature was initially raised to 230°C to allow nucleation and growth of the particles. A previous calibration was used to determine the optimal size of the QDs. The solution was then cooled to room temperature to quench the reaction.

Without any further purification, the same reaction vessel was used to grow ZnS shell $CuInS_2$ core QDs. The shell precursors solution was prepared separately in a three-neck flask before injection by mixing 2 mmol of $Zn(St)_2$ 2.5 mL of OA, 2.5 mL of DDT and 5 mL of ODE. The shell precursor solution was degassed for 30 min at room temperature and then at 100°C for 1h, until complete dissolution of $Zn(St)_2$ and finally filled with nitrogen and collected with a syringe. After heating the core solution to 220°C the shell precursor solution was continuously injected with a syringe pump for 4 hours. Finally the crude CuInS2/ZnS QDs solution was washed with a 1:2 hexane : ethanol solution and centrifuged at 3000 rpm for 5 minutes for three times.

Fabrication of $CuInS_2$/ZnS-based LSC - An industrial cell casting process was used to fabricate the PMMA waveguide by bulk polymerization. The process was characterized by two steps. First, the so-called "syrup" was prepared: the monomer, purified through a basic alumina column, was heated to 80 °C in a beaker. When the MMA temperature stabilized, AIBN (100 ppm w/w with respect to MMA) was added. At this point, pre-polymerization (an exothermic process) took place and the monomer temperature was increased to the MMA boiling temperature (95 °C). When the monomer reached the stage of strong boiling, the syrup was quenched. In the second stage, the prepolymer was degassed by four freeze-pump-thaw cycles to remove oxygen and bring in an argon

atmosphere, and then mixed with the dispersion of QDs in MMA with lauryl peroxide (400 ppm by weight with respect to MMA) (10% by weight with respect to the syrup) described above. The viscosity of the liquid was then introduced into the mold, where the polymerizing reaction took place. The casting mold was made up of two glass plates that were sealed with a polyvinyl chloride (PVC) gasket (in order to maintain the inert atmosphere) and were clamped together. To accommodate the shrinkage of the polymer plate during the polymerization process, the clamps contained springs. The mold was placed in a water bath at 55 °C for 48 h. Finally, the plate was post-cured in an oven at 115 °C overnight.

Monte-Carlo ray tracing simulations of QDs LSC - The theoretical analysis of the efficiency of an ideal free from scattering and reabsorption losses LSC was performed via a Monte Carlo ray tracing technique. All LSC dimensions by far exceed wavelengths of photons within the energy range of interest (500 - 700 nm). Therefore, propagation of a photon within the LSC could be modeled as a propagation of a ray (beam) subject to refraction and reflection at the air-LSC interfaces according to Fresnel laws. The stochastic nature of the simulation is reflected in the fact that the ray is not split upon reaching an interface but rather either transmitted or reflected with the probabilities proportional to respective energy fluxes given by Fresnel laws. The dependence of these probabilities on the state of polarization of the incident ray (e.g., s- or p-polarized) was also taken into account. A specific event (i.e., transmission or reflection) is chosen according to random drawing. Inside the LSC material, for each photon, the inverse transform sampling method is applied to randomly generate the length of the optical path before this photon is absorbed by an emitter. Path lengths follow the exponential attenuation law determined by the wavelength-dependent absorption coefficient, α(λ), related to the absorption cross-section, $\sigma(\lambda)$, and the QDs concentration, N_{QD}, by $\alpha(\lambda)= N_{QD}\,\sigma(\lambda)$. Since the mean path length, given by the inverse absorption coefficient is always much greater than the average distance between the emitters, there was no need to keep track of an explicit position of each emitter; therefore, the nanocomposite can be considered within the effective medium approach, that is, as a uniform material with the absorption coefficient defined above. Once a photon is absorbed, the subsequent fate of the excitation is again determined by the Monte Carlo sampling assuming unity PLQY. The direction of re-emitted photons is distributed uniformly across the 4π sphere and the re-emission wavelength is determined using the rejection sampling applied to a representative PL spectrum having no overlap with the absorption profile. Within these assumptions, the ultimate fate of each photon is escape from the LSC via one of its faces. A single-ray Monte Carlo simulation is typically repeated 10^3 - 10^6 times to have a proper statistical averaging.

LSC Characterization - The LSC was characterized using a 15 AMG calibrated solar simulator (ABET 2000) with power density P = 100 mW cm^{-2} (1.5 AM Global). The LSC was equipped with Si-based PV solar cells coupled to the four edges using an optical glue. The electrical response was measured with a Keithley 2602 source meter. Brown flour with different grain size was used to simulate dust deposits. Wetting and dried residues experiments were performed using pure water and dried NaCl water solution, respectively.

Optical characterization – Optical absorption spectra were recorded by a Varian Cary 50 spectrometer at normal incidence. Steady-state PL measurements were performed exciting the samples with a 405 nm pulsed diode laser (Edinburgh Inst. EPL 405, 40 ps pulse width) and collecting with a TM-C10083CA Hamamatsu Spectrometer. The LSC transmittance was measured with a calibrated solar simulator and a photodiode (Thorlabs PM100D – sensor S120VC). The average transmittance was averaged over five different spots of the LSC.

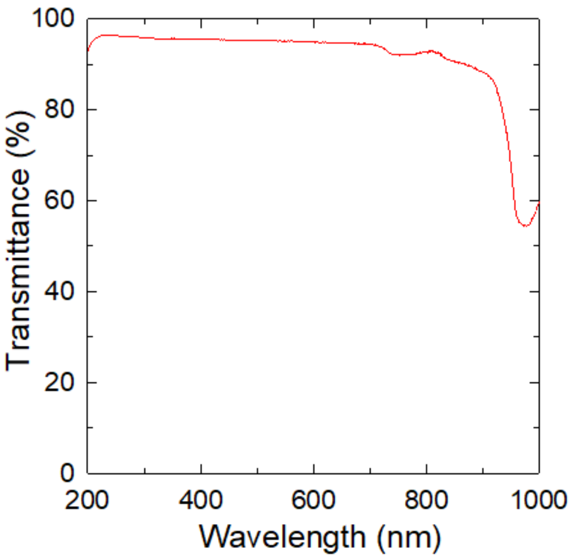

Figure S1: Transmittance spectrum of distilled water collected from a 10 mm optical path Suprasil Quartz cuvette.
